# Analysis of COVID-19 Collective Irrationalities Based on Epidemic Psychology

**DOI:** 10.3389/fpsyg.2022.825452

**Published:** 2022-03-21

**Authors:** Hua Luo, Yu Ren

**Affiliations:** ^1^Department of Orthopedics, Taizhou Hospital of Zhejiang Province Affiliated to Wenzhou Medical University, Taizhou, China; ^2^Department of Pharmacy, Taizhou Hospital of Zhejiang Province Affiliated to Wenzhou Medical University, Taizhou, China

**Keywords:** COVID-2019, epidemic psychology, collective irrationalities, rumors, scarcity psychology

## Abstract

As the SARS-CoV-2 virus swept the world in late 2019, it has brought widespread fear, some suspicion, and degrees of stigma. In the shadow of the COVID-19 pandemics, a series of collective irrationalities such as panic buying, protest marches against vaccines, and pandemic stigma occurred. This phenomenon is inseparable from the spread of rumors about the epidemic. The advent of social media has radically changed the way we consume information and form opinions and made a flood of digital misinformation becoming ubiquitous. The diffusion of false rumors affects the public’s perception of reality and disrupts the prevention of the epidemic. This paper analyzes the COVID-19 collective irrationalities from epidemic psychology to provide a new reference view for overcoming psychological problems related to COVID-19.

## Introduction

In December 2019, coronavirus disease 2019 (COVID-2019) occurred in Wuhan, was spread to the whole country and even the entire world, and was identified as a public emergency of international concern by WHO. However, in the strict sense, while the spread began then, the speed and breadth of widespread infection were not recognized until early 2020. Now, much of the world is gripped by Omicron mutant strains. The WHO considered the current COVID-19 outbreak a global pandemic based on assessments (Wang et al., 2020). About 250.2 million confirmed cases and over 5.1 million confirmed deaths were reported to WHO till November 09, 2021 (World Health Organization, 2021). COVID-19 is highly infectious, widely spread, and rapidly progressing, posing a significant threat to some populations’ physical and mental health. There are collective irrational phenomena in the shadow of the COVID-19, such as panic buying, protest marches against vaccines, and pandemic stigma. This phenomenon is inseparable from the spread of rumors about the epidemic. The advent of social media has radically changed the way we consume information and form opinions and made a flood of digital misinformation ubiquitous. Rumors and questionable information spreading can strongly influence people’s behavior and alter the effectiveness of the countermeasures deployed by governments. The term *infodemic* has been coined to outline the perils of misinformation phenomena during the management of disease outbreaks (Zarocostas, 2020). It could even speed up the epidemic by influencing and fragmenting social response (Kim et al., 2019).

Thirty years ago, Philip Strong philosophically defined the reaction to major infectious diseases as a unique psychosocial form, called *epidemic psychology* (Strong, 1990). Philip believes that an outbreak of fatal epidemics seems to be followed by fear, panic, suspicion, and pandemic stigma. It is no exception that the COVID-19 pandemic has brought widespread fear, some suspicion, and degrees of stigma. Massive digital misinformation is becoming pervasive in online social media, to the extent that the World Economic Forum (WEF) is one of the main threats to our society (Howell, 2013; Quattrociocchi, 2016). The advent of the current pandemic has brought forth conspiracy theorists and panic claims. In the early days of the outbreak, the Internet was rife with stigmatized and absurd rumors that the epidemic was caused by human consumption of bats, and conspiracy theorists attributed SARS-COV-2 to leaks from biochemical laboratories.

A social psychology study from the University of Kent said that while there was no evidence that people exposed to the Internet were more likely to believe conspiracy theories, the Internet made the conspiracy theorists more convinced. Coincidentally, Gustave Le Bon described in his work *The Crowd: A Study of the Popular Mind*: A lie repeated a thousand times does not become truth, but it will convince the crowd that it is a scientific truth (Gustave, 2002). The way people communicate and provide information on the Internet can reinforce their original views. For example, algorithms make it easier for them to receive information similar to what they were previously viewing constantly, and choosing which online communities to communicate with is a process of “choosing whom to listen to and whom to block by default.” These phenomena do not impact people who do not already believe in conspiracy theories, but they strengthen conspiracy theorists’ views and cognition. In such a context, individuals can be uninformed or misinformed. Moreover, the spread of misinformation might be complicated to detect and correct. People are more likely to trust the information consistent with their beliefs (Meade and Roediger, 2002; Centola, 2010; Quattrociocchi et al., 2014), ignore dissenting, and form polarized groups around shared information (Del Vicario et al., 2016). It comes at the expense of the information’s quality and leads to the proliferation of biased narratives fomented by unsubstantiated rumors, mistrust, and paranoia (Del Vicario et al., 2016). Many mechanisms cause false information to gain acceptance, which in turn generate false beliefs once adopted by an individual, they are highly resistant to correction (Kelly and Weeks, 2013).

The term *infodemic* has been coined to outline the perils of misinformation phenomena during the management of disease outbreaks since it could even speed up the epidemic process by influencing and fragmenting social response (Kim et al., 2019). Numerous studies revealed that young people are psychologically more vulnerable and prone to depression than the elderly due to the isolation measures during the coronavirus pandemic, which affected routine work, increased economic pressure, and hindered social activities (Jia et al., 2020). Moreover, excessive exposure to information related to the outbreak on social media may also trigger adverse effects (Zhao, 2020). The Internet is an abundant environment for the massive diffusion of unverified rumors, and the spreading of rumors may foster panic. So-called panic buying, which did arise in the United Kingdom, is a behavior pattern at the beginning of lockdown. Moreover, this is not just an accidental phenomenon. For example, in the early stages of the outbreak, the media reported a rumor about the complete lockdown of Wuhan to prevent rumors of a pandemic. As a result, people drove overcrowded supermarkets to stockpile food. Many people drove to escape from Wuhan to other regions before the lock-down was put in place, disrupting the government initiative aimed to contain the epidemics and potentially increasing contagion. Eventually, the panic subsided amid clarify in the official media.

Moreover, panic brought by rumors also can cause us to overestimate the risk of low-probability events. In this case, on the one hand, panic has contributed to our overestimation of things like “food is not available.” On the other hand, the risk of infection has been overestimated. We see that 250.2 million people have been confirmed, and more than 5.1 million people have died (November 09, 2021). The vast numbers give us the impression that many people are infected, but mortality is not when expanding horizons to include the whole world. Consider that during the first wave, the mortality rate in the United Kingdom was over 15% (deaths/total new cases), whereas now it is 1.5%. Similarly, Italy’s mortality rate has dropped to 2.8%. However, Mexico’s mortality rate is 7.6%, and Bolivia’s is 3.7%. In the first wave, the United States mortality rate was 4.3%, dropping to 1.62% (World Health Organization, 2021). It is evident that after the initial horror of COVID-19, these outbreaks are no longer as deadly. COVID-19, while still terrifying in the context of this strict containment, now, at least in some cases, it will confront in familiar ways: “COVID-19 is back, and we must seal off the city.” Of course, this is based on the premise of comprehensive protection.

Since the outbreak of COVID-19, people’s social life has changed. Under the new situation, coronavirus pandemic prevention and control have become routine work. After strict prevention and power in the early stage of the coronavirus pandemic, China’s whole situation displays a good trend (Dong et al., 2020). Also, an increasing number of variants now recognized worldwide emphasize consideration of travel restrictions. However, as coronavirus pandemic prevention and control becomes routine, the “extraordinary measures” such as lockdown, isolation, and quarantine will eventually face a “ceiling.” The global pandemic of COVID-19 has never stopped, and the best approach is to establish herd immunity through vaccines, but this is a gradual process. The vaccine’s birth has brought hope and confidence to the public as expected, but it has also brought a series of problems. Since misinformation influences individuals’ beliefs (e.g., risk perceptions), it may also influence vaccination attitudes (Betsch et al., 2015). Vaccine hesitancy, regarded as one of the 10 most significant global health threats today, is also a clear threat to COVID-19 control. New data show that willingness to take a COVID-19 vaccine is far from universal (The Lancet, 2020). Rapid vaccine development may also confuse public perception and concern about the safety and efficacy of anti-COVID-19 vaccines once approved. Recently, a Facebook group called Le Convoy de la liberté gained more than 320,000 followers. Protesters gathered in a “freedom convoy” in Paris to block the main road leading into the city to show their opposition to the vaccine pass measure. This campaign confirmed that fake news and inaccurate information might spread faster and broader than fact-based news. Although the neutralization activity of serum samples from vaccinated people and recovered patients decreased by about two times, it is still effective against the mutant strain. In addition, recent reports of imported cases in China suggest that vaccination reduces the risk of infection and the rate of severe cases in vaccinated patients to some extent.

How to curb the spread of rumors and false information? Le Pen claimed that once an individual is integrated into a group, his personality will be annihilated, and the group’s thoughts will occupy an absolute dominant position. Meanwhile, the group’s behavior will also be characterized by a rejection of dissent, extreme, emotional, and low intelligence, which will have a destructive impact on society. Therefore, the psychology of the masses is not rational at all. They are a group of crazy, impulsive, paranoid, blind, fanatical, and easily agitated unconscious mediocrity, namely the mob. There is nothing so common as the denunciation of the masses today of what they praised yesterday. Therefore, accessible discussion of different views will not find the truth but will numb people. However, the theory of “free market of opinions” holds that group rationality truth becomes clearer when argued over, free expression of ideas is a political right, and discussion of fallacies clarifies fact. John Milton first developed this theory in England in his book Areopagitica (John, 2019). He believed that falsehood and truth must be equally disseminated. The British philosopher John Stuart Mill believed that anyone who attempts to use authority’s power to suppress speech freedom is unreasonable. If pressed statements are correct, not only trampled by suppressing political rights, and the oppressor itself was also deprived of the opportunity to mistake the truth. If oppressed speech or thought is wrong, the chance for the truth to be revealed in the open contest between truth and error is also lost. Therefore, suppressing people’s speech or thoughts so that they cannot be freely expressed is bound to plunder the intelligence of individuals and even the entire human race.

Disseminating rumor rebuttal content on social media is vital for rumor control and disease containment during public health crises. It is a thorny issue, especially in the Internet information age. When some emergencies occur, online rumors can easily cause panic among the public and affect social stability, and the harm is apparent. It is far from enough to rely only on the gentleman’s “rumors stop with the wise” mentally. The government should actively lead public opinion. Detected online rumors early, controlled effectively, and eliminated them in the bud. In addition, the emergency response mechanism should be improved. Once there were rumors, coordinate relevant departments to quickly deliver the truth to the public as soon as possible. Raise the cost of rumors production and transmission and let those who intended to achieve illegal personal purposes spread rumors through the network prohibitive. For example, google news decided to flag fact-checked information and penalize fake news; others propose using blocklists of sources to limit their spread automatically. In a word, the more open the society is, the less room there is for talks to survive. From this perspective, the openness, transparency, and timeliness of information are the basic principles for dealing with all public events, including online rumors.

In addition, repeated outbreaks are indeed chronic stress on public mental health. Therefore, while preventing and controlling COVID-19, attention to the public’s psychological state and health should not be ignored, and COVID-19-related mood management and social support should be provided to relieve psychological problems in the general public. It is remarkable that with the coronavirus pandemic, the world must function as one community to maximize control. Public protective behavior and cognition play a crucial role in controlling the outbreaks of pandemics and mental state. At least on the surface, epidemic psychology seems to be a universal human trait. However, different societies may have very other preferences, different types of structures, and various tools at their disposal when dealing with the threat to public order. Considering that COVID-19 has “integrated” deeply into human society, we may have to continue to live in a coronavirus pandemic environment in the future. Then how to carry on regular production and life in the coronavirus pandemic environment needs to be explored gradually. Society needs to put aside the abnormal attitude to realize the normalization of coronavirus pandemic prevention and control, let everything happen naturally, start a new life in the new normal. Finally, it is worth emphasizing a little that the price of health is eternal vigilance.

## Data Availability Statement

The original contributions presented in the study are included in the article/supplementary material, further inquiries can be directed to the corresponding author.

## Author Contributions

HL performed the literature retrieval and drafted the article. YR conceived the project and provided suggestions to improve it. All authors contributed to the article and approved the submitted version.

## Conflict of Interest

The authors declare that the research was conducted in the absence of any commercial or financial relationships that could be construed as a potential conflict of interest.

## Publisher’s Note

All claims expressed in this article are solely those of the authors and do not necessarily represent those of their affiliated organizations, or those of the publisher, the editors and the reviewers. Any product that may be evaluated in this article, or claim that may be made by its manufacturer, is not guaranteed or endorsed by the publisher.

## References

[ref1] Betsch C. Bohm R. Chapman G. B. (2015). Using behavioral insights to increase vaccination policy effectiveness. Policy Insights Behav. Brain Sci. 2, 61–73. doi: 10.1177/2372732215600716

[ref2] Centola D. (2010). The spread of behavior in an online social network experiment. Science 329, 1194–1197. doi: 10.1126/science.1185231, PMID: 20813952

[ref3] Del Vicario M. Bessi A. Zollo F. Petroni F. Scala A. Caldarelli G.. (2016). The spreading of misinformation online. Proc. Natl. Acad. Sci. U. S. A. 113, 554–559. doi: 10.1073/pnas.1517441113, PMID: 26729863 PMC4725489

[ref4] Dong L. Hu S. Gao J. (2020). Discovering drugs to treat coronavirus disease 2019 (COVID-19). Drug Discov. Ther. 14, 58–60. doi: 10.5582/ddt.2020.01012 32147628

[ref5] Gustave L. B. (2002). The Crowd: A Study of the Popular Mind. New York, USA: Dover Publications Press.

[ref6] Howell W. (2013). Digital Wildfires in a Hyperconnected World. Cologny, Switzerland: World Economic Forum.

[ref7] Jia R. Ayling K. Chalder T. Massey A. Broadbent E. Morling J. R.. (2020). Young people, mental health and COVID-19 infection: the canaries we put in the coal mine. Public Health 189, 158–161. doi: 10.1016/j.puhe.2020.10.018, PMID: 33249392 PMC7598559

[ref8] John M. (2019). Areopagitica. Wentworth, Australia: Wentworth Press.

[ref9] Kelly G. Weeks B. (2013). “The promise and peril of real-time corrections to political misperceptions.” in *Proceedings of the 2013 Conference on Computer Supported Cooperative Work*; February 23, 2013 (ACM, New York).

[ref10] Kim L. Fast S. M. Markuzon N. (2019). Incorporating media data into a model of infectious disease transmission. PLoS One 14:e0197646. doi: 10.1371/journal.pone.0197646, PMID: 30716139 PMC6361417

[ref11] Meade M. L. Roediger H. L. 3rd. (2002). Explorations in the social contagion of memory. Mem. Cogn. 30, 995–1009. doi: 10.3758/bf03194318, PMID: 12507365

[ref12] Quattrociocchi W. (2016). How Does Misinformation Spread Online? Geneva, Switzerland: World Economic Forum.

[ref13] Quattrociocchi W. Caldarelli G. Scala A. (2014). Opinion dynamics on interacting networks: media competition and social influence. Sci. Rep. 4:4938. doi: 10.1038/srep04938, PMID: 24861995 PMC4033925

[ref14] Strong P. (1990). Epidemic psychology: a model. Sociol. Health Illn. 12, 249–259. doi: 10.1111/1467-9566.ep11347150

[ref15] The Lancet (2020). COVID-19 vaccines: no time for complacency. Lancet 396:1607. doi: 10.1016/s0140-6736(20)32472-7, PMID: 33220729 PMC7834228

[ref16] Wang C. Horby P. W. Hayden F. G. Gao G. F. (2020). A novel coronavirus outbreak of global health concern. Lancet 395, 470–473. doi: 10.1016/s0140-6736(20)30185-9, PMID: 31986257 PMC7135038

[ref17] World Health Organization (2021). WHO Coronavirus Disease (COVID-19) Dashboard. Geneva: World Health Organization. Available at: https://covid19.who.int/ (Accesed November 9, 2021).

[ref18] Zarocostas J. (2020). How to fight an infodemic. Lancet 395:676. doi: 10.1016/s0140-6736(20)30461-x, PMID: 32113495 PMC7133615

[ref19] Zhao N. (2020). Social media use and mental health during the COVID-19 pandemic: moderator role of disaster stressor and mediator role of negative affect. Int. J. Ment. Health Addict. 2, 1019–1038. doi: 10.1111/aphw.12226 PMC7536964 32945123

